# In-hospital costs of community-acquired colonization with multidrug-resistant organisms at a German teaching hospital

**DOI:** 10.1186/s12913-018-3549-0

**Published:** 2018-09-26

**Authors:** Sabine Engler-Hüsch, Thomas Heister, Nico T Mutters, Jan Wolff, Klaus Kaier

**Affiliations:** 1 Institute for Medical Biometry and Statistics, Faculty of Medicine and Medical Centre – University of Freiburg, Freiburg, Germany; 2 Institute for Infection Prevention and Hospital Epidemiology, Medical Centre – University of Freiburg, Faculty of Medicine, University of Freiburg, Freiburg, Germany; 3 Department of Psychiatry and Psychotherapy, Faculty of Medicine and Medical Centre – University of Freiburg, Freiburg, Germany

**Keywords:** Multi-drug resistance, Colonization, G-DRG, Hospital cost, Reimbursement

## Abstract

**Background:**

Antibiotic resistance is a challenge in the management of infectious diseases and can cause substantial cost. Even without the onset of infection, measures must be taken, as patients colonized with multi-drug resistant (MDR) pathogens may transmit the pathogen. We aim to quantify the cost of community-acquired MDR colonizations using routine data from a German teaching hospital.

**Methods:**

All 2006 cases of documented MDR colonization at hospital admission recorded from 2011 to 2014 are matched to 7917 unexposed controls with the same primary diagnosis. Cases with an onset MDR infection are excluded from the analysis. Routine data on costs per case is analysed for three groups of MDR bacteria: Methicillin-resistant *Staphylococcus aureus (*MRSA), vancomycin-resistant *enterococcus* (VRE), and multidrug-resistant gram-negative bacteria (MDR-GN). Multivariate analyses are conducted to adjust for potential confounders.

**Results:**

After controlling for main diagnosis group, age, sex, and Charlson Comorbidity Index, MDR colonization is associated with substantial additional costs from the healthcare perspective (€1480.9, 95%CI €1286.4–€1675.5). Heterogeneity between pathogens remains. Colonization with MDR-GN leads to the largest cost increase (€1966.0, 95%CI €1634.6–€2297.4), followed by MRSA with €1651.3 (95%CI €1279.1–€2023.6), and VRE with €879.2 (95%CI €604.1–€1154.2). At the same time, MDR-GN is associated with additional reimbursements of €887.8 (95%CI €722.1–€1053.6), i.e. costs associated with MDR-colonization exceed reimbursement.

**Conclusions:**

Even without the onset of invasive infection, documented MDR-colonization at hospital admission is associated with increased hospital costs, which are not fully covered within the German DRG-based hospital payment system.

**Electronic supplementary material:**

The online version of this article (10.1186/s12913-018-3549-0) contains supplementary material, which is available to authorized users.

## Background

Antibiotic resistance is a major challenge in the management of infectious diseases [1]. Treatment of healthcare-associated infections (HAIs), a frequent adverse event in health care delivery, is complicated when the causative pathogen is resistant. However, antimicrobial resistance (AMR) also impacts patients who do not become infected. Admission to a hospital while carrying multidrug-resistant (MDR) bacteria can be associated with prolongation of stay or increased medical costs [2–4]. MDR bacteria are organisms that are insusceptible to several classes of antibiotics.

Even adjusted for severity of underlying illnesses, length of stay (LOS) is significantly increased if a patient is colonized with a MDR bacteria [5]. Many factors besides morbidity can influence LOS in colonized patients in comparison to non-colonized patients: Patients colonized with an MDR are often put in spatial isolation, which can have negative psychological effects on the patients, affecting clinical outcomes [6].

The way isolation of colonized patients is implemented in the hospitals’ daily routine might also play a role: Anecdotal evidence suggests that hospitals or clinical departments tend to schedule diagnostics for colonized patients at the end of the day. If an emergency leads to an unscheduled patient having to be diagnosed immediately, the MDR patient, as the last patient on the regular schedule, is the patient most likely to be deferred to the next day. Such factors might also contribute to the longer LOS in colonized patients.

Depending on the pathogen, colonization can persist for months or even years, if untreated [7], potentially influencing costs over multiple hospital stays.

Quantifying the costs of colonization without infection therefore is an important piece in the overall picture of cost of MDR, and precise measurement of these costs is vital to efficiently allocate hospital resources to most effectively control them [8]. These costs include expenses for pathogen detection, infection control measures, and loss of reimbursement associated with bed closures due to patient isolation [9]. Costs can differ between MDR organisms such as Methicillin-resistant *Staphylococcus aureus (*MRSA), Vancomycin-resistant *Enterococcus* (VRE), or multidrug-resistant gram negative bacteria (MDR-GN), [5, 10]. MDR-GN include multidrug-resistance in *K. pneumoniae*, A. baumannii, *P. aeruginosa*, Enterobacter spp., *E. coli* and other pathogens.

Using a single-centre, matched case-cohort design, we quantify the economic burden of patients admitted to the hospital while colonized with MRSA, VRE, or MDR-GN, focusing on the inclusion of risk adjustment scores as well as accounting for procedure-related fixed costs.

## Methods

### Data

The data is obtained from the University Medical Centre Freiburg (UMCF), a tertiary care teaching hospital with some 1600 beds. All cases from January 2011 to December 2014 are included. Complete routine data of 185,348 inpatient cases from different wards including intensive care units (ICU) are available. Only patients older than 17 years were selected. The dataset includes detailed information about individual patient characteristics such as an individual unique identifier for each admission, age, sex, main and secondary diagnoses, costs and reimbursement. All patient records were stripped of identifying information prior to release to the researchers in accordance with German data protection law.

In-hospital treatments in Germany are generally reimbursed through predetermined lump-sums based on diagnosis related groups (DRGs). The criteria for grouping DRGs include the patients’ diagnoses, sex and age, treatment procedures, and comorbidities, among others. Hospitals receive additional reimbursement for every day that a patient stays above the upper length of the stay threshold to compensate for unusually long stays [11]. These daily surcharges are, however, much lower than the mean reimbursement per day below this threshold, and designed to not entirely cover additional variable costs to create incentives to reduce length of stay. Additional reimbursement is made for cases with very severe illnesses necessitating complex intensive care treatments [12].

About 250 hospitals are tasked with generating detailed real-life cost figures by recording the individual services delivered to each patient. Until 2016, the sample was made up entirely of hospitals that had volunteered for this task. Since then the sample has been expanded with hospitals randomly selected from those hospitals whose patient and procedure profiles had previously been underrepresented to improve the representativeness of the sample. The cost numbers conform to a standardised costing system developed by the Institute for the Payment system in Hospitals (InEK), the authority responsible for reimbursement rates [11]. Reimbursement of inpatient cases is therefore informed by real cost data. Direct costs, which are mandatory for implants, blood products or drugs etc., are based upon documented utilization. Overhead costs and costs on primary cost units on the other hand are based upon key cost drivers, i.e. time on ward or in the ICU or operating room. Indirect cost units such as on demand medications or dressings are allocated to primary cost units and excluded unless they are relevant for the corresponding DRG [13–15].

Main and secondary diagnoses are coded with the International Classification of Diseases 10th revision, German Modification (ICD-10-GM).

Microbiological data was obtained from the Institute for Infection Prevention and Hospital Epidemiology on the three most relevant groups of MDR bacteria MRSA, VRE and MDR-GN.

### Community-onset cases

Community-onset cases of colonization are identified using the timestamps from the microbiological data. The threshold is a pathogen detection < 48 h after admission. A detection more than 48 h after admission leads to exclusion from further analyses, as these are considered hospital-acquired [16]. In a second step all onset infections associated with a resistant pathogen are excluded. Although this step leads to a study population containing on average more healthy individuals, it is necessary to isolate the costs of colonization, as infection in itself leads to substantial increases in costs and reimbursements. Moreover, transplantations are excluded, since transplantations involve two patients, but are only reimbursed by the health insurance of the recipient such that costs are assigned to both donor and recipient, while reimbursements are only attributed to the recipient. Inpatient cases with documented MDR-colonisation at hospital admission are compared to controls never infected or colonized with a MDR organism.

### Matching

Each of the positive cases (*n* = 2006) is randomly matched with up to four controls (*n* = 7917) within the same primary diagnosis (4 digit, ICD-10). Eligible controls are required to not have a positive resistant pathogen status during their stay. For a few cases there were less than four controls satisfying the matching criteria available. Again, patients colonized > 48 h after admission, patients with onset infections and transplant patients were excluded from the pool of potential controls. The primary diagnosis matching is used since in the G-DRG payment system, costs and reimbursements are highly clustered within main diagnosis groups, as most costs are disease- or procedure-related. This within-main-diagnosis approach prevents the comparison of controls with cases with different fixed costs unrelated to the colonization with resistant pathogens. Multivariate analyses are conducted to include additional potential confounders such as age, sex and comorbidities.

### Risk adjustment

For risk adjustment, the Charlson comorbidity index (CCI) is applied [17]. The CCI is a weighted index consisting of 19 comorbid conditions. The score was adjusted as described by Quan et al. [18] to comply with the ICD-10 systematic and is a widely used tool to control for underlying differences in comorbidities when evaluating attributable mortality, length of stay or costs in patients.

### Regression model

For the multivariate analyses, a generalized linear model (GLM) is chosen, to account for the right skewed distribution of health care cost data and reduce sensitivity to outliers [19, 20]. For the outcome (InEK-)costs and reimbursements, a log link and a gamma distribution were chosen, based on the results of Modified Park Tests. The models include the main diagnosis groups as fixed effect as well as age, age^2^, sex, and the CCI as continuous and categorical covariates, respectively. Two different models are estimated for each outcome, first using the aggregated binary variable, indicating a positive pathogen status, and second the three pathogens separately. All models use robust standard errors. As log links are used, exponentiated coefficients represent the multiplicative increase in the costs (or reimbursements) from a one-unit increase in the respective independent variable. Because GLMs focus inference on the overall marginal mean, predictions of mean costs (or reimbursements) are estimated from the model [21]. Computation of standard errors or confidence intervals for the additive difference in means is obtained using the “margins” command in Stata [22], which computes standard errors using the delta method.

For the statistical analysis Stata Version 14.1 (Stata Corp, College Station, Texas, USA) is used.

## Results

### Patient population

Table 1 presents the descriptive statistics for cases and unexposed controls. Due to the within-main-diagnosis matching process, 7917 controls are elected for the multivariate analysis from the pool of 183,378 possible controls. As can be seen, the selected actual controls are much more similar to the cases in terms of relevant characteristics such as cost, illness severity (CCI) and age as a result of the matching process.

**Table 1:** Descriptive statistics

	1		2		3	
	Possible controls		Actual controls		Community acquired cases	
	Mean/%	SD	Mean/%	SD	Mean/%	SD
Number of unique main diagnoses	4764		644		644	
Cost per case, in €	4106.53	5819.72	5504.37	8125.41	6706.44	8350.41
Reimbursement per case, in €	4230.74	5492.32	5438.42	7023.13	6222.75	7165.54
Length of hospital stay, in days	6.80	6.91	8.93	9.21	10.28	9.55
Age, in years	57.35	18.75	60.76	17.38	62.14	16.48
Charlson comorbidity index	1.79	2.72	3.38	3.36	4.34	3.44
In-hospital mortality, %	1.33	11.45	2.43	15.38	3.74	18.98
Female, %	49.35	50.00	44.47	49.70	42.47	49.44
N	185,348		7917		2006	

Notes: Column 1 shows all available possible controls in the dataset. Column 2 shows controls chosen for the regression model after the matching process. Column 3 shows cases eligible for the regression model

Descriptive statistics regarding the differences between the three resistant pathogens MRSA, VRE and MDR-GN are shown in Additional file 1. Co-colonization with more than one pathogen is possible. Heterogeneity between the pathogens is visible, with VRE being associated with the largest costs and reimbursements. Since the results are unadjusted for possible confounding factors, the effects are most likely driven by comorbidities and/or advanced age.

### Adjusted statistical analysis

Table 2 shows the results of the regression for the outcomes cost and reimbursement, at first for all community-acquired cases combined, then separately for each of the three pathogens. In order to interpret the estimation results, the coefficients were transformed to present the percentage increase of the variable of interest [23]. Below the estimates, marginal effects are calculated. Being colonized with a resistant pathogen increases costs per case by 26% or 1500€ compared to controls. The cost effects differ between the pathogens. While MDR-GN leads to the largest cost increase, of nearly 2000€ per case, MRSA is associated with a cost increase of over 1600€ per case, followed by VRE with nearly 900€ all other things equal.

**Table 2:** Regression results for costs and reimbursements

	(1)	(2)	(3)	(4)
	Costs	Costs	Reimbursements	Reimbursements
Community-onset cases	0.256***		0.158***	
	[0.224,0.289]		[0.129,0.188]	
*Marginal effect in €*	1480.9***		887.8***	
	[1286.4,1675.5]		[722.1,1053.6]	
MRSA		0.286***		0.212***
		[0.222,0.350]		[0.154,0.270]
*Marginal effect in €*		1651.3***		1188.6***
		[1279.1,2023.6]		[861.1,1516.1]
VRE		0.152***		0.0583***
		[0.105,0.200]		[0.0162,0.100]
*Marginal effect in €*		879.2***		326.5***
		[604.1,1154.2]		[90.52,562.5]
MDR-GN		0.341***		0.236***
		[0.284,0.397]		[0.186,0.286]
*Marginal effect in €*		1966.0***		1320.9***
		[1634.6,2297.4]		[1038.3,1603.4]
CCI	0.0533***	0.0541***	0.0433***	0.0440***
	[0.0469,0.0597]	[0.0477,0.0605]	[0.0377,0.0489]	[0.0384,0.0496]
*N*	9923	9923	9923	9923

Note: All Models are estimated with GLM regressions. Coefficients are exponentiated and substracted by 1. All columns include within main diagnosis fixed effect and are controlled for age, age^2^ and sex. CCI: Charlson comorbidity index. 95% confidence intervals in brackets

*p < 0.1, **p < 0.05, ***p < 0.01

**Fig. 1:**
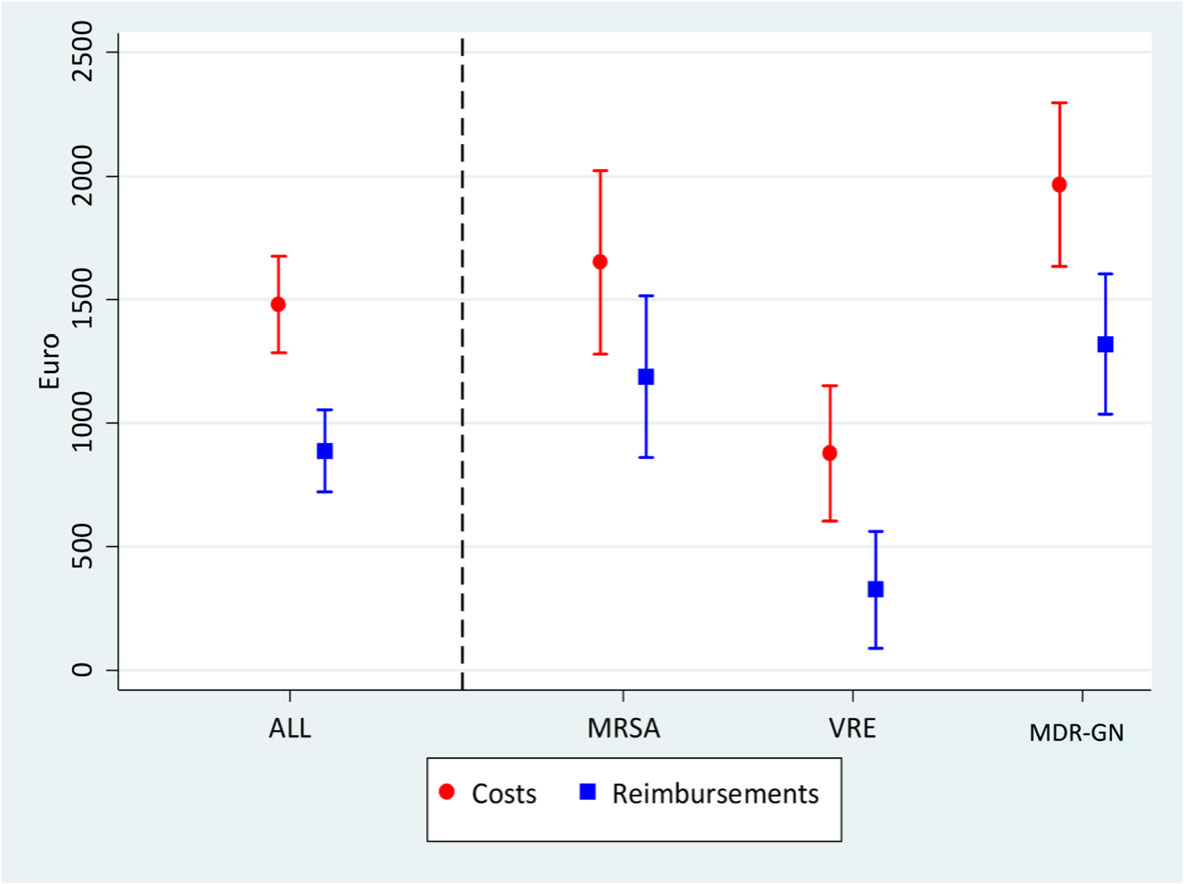
Additional costs of MDR-colonised patients in comparison to non-colonised controls. Notes: Risk adjusted estimates with 95% confidence intervals as calculated in Table 2

Results for the outcome reimbursement show a similar pattern. Patients colonized with a resistant pathogen accrue additional reimbursements of around 887€ per case or 16% more than controls. Cases with the highest cost estimates have larger reimbursements, as visualized in Fig. 1, although the difference is negative for all three pathogens. We find cost increases for MDR-GN cases of about 1300€ compared to unexposed controls, 1200€ for MRSA cases, and about 300€ for VRE cases.

As shown in Fig. 1, additional costs associated with colonizations are higher than the compensation payment.

## Discussion

We find that cases colonized with MDR pathogens are associated with additional costs even without the onset of an infection. Even though these cases are asymptomatic carriers, they incur a considerable financial burden. Additionally, our study focused on community-onset cases of colonization, estimating the financial burden of hospitals due to the influx of MDR cases from the community. Even excluding transmissions occurring in the hospital, costs due to colonization are considerable and exceed reimbursement, in effect penalizing hospitals for events outside their control. These extra costs may be due to measures to prevent the spread of pathogens such as single room isolation, but can also indicate higher treatment cost [24].

Second, the results show that it is important to account for possible heterogeneity between different pathogens, as the large differences between our findings for MRSA, VRE and MDR-GN attest. This may be explained by the observation that some pathogens are more commonly found in specific tissues than others, so that the pathogens differ regarding their detectability via screening and testing [25]. It is, for example, easier to screen for skin colonizing MDR organisms than those colonizing the gut. Additionally, decolonisation measures are sometimes used for some pathogens, such as MRSA [26]. Decolonization measures would incur cost through the expenses for the measures themselves, but, if successful, reduce costs for the remainder of that patient’s hospital stay, since the decolonized patient would no longer have to be isolated [27]. This may explain some of the differences in the cost of MRSA compared to MDR-GN or VRE, for which no decolonisation regimens yet exist.

Finally, it is sometimes difficult to assess the risk of subsequent infections by the colonising MDR. For example, in case of asymptomatic colonisation of the respiratory tract by a MDR-GN, physicians still might choose to treat the patient with antibiotics to prevent subsequent pneumonia. Treatment costs would increase, and possibly even LOS if patients are kept longer in the hospital for observation.

Limitations of this study include the definition of the variable of interest, community-onset resistant colonization. It is not possible to distinguish between genuine community-acquired and previous healthcare-associated colonization from previous visits to health care facilities or nursing homes. Previous studies found a correlation between colonization and previous hospitalization [28, 29]. Unfortunately, our routine data does not provide information about previous visits to such facilities. It is possible that the variable is driven by unobserved comorbidities, as it can be hypothesized that a patient being transferred from a health care facility is on average not as healthy as a patient transferred from home, keeping age and sex constant. Following this assumption, the variable identified in this study would thus not directly measure the economic burden of colonization cases, but rather work as an additional indicator for unobserved comorbidities.

However, our matching of controls and MDR carriers included the CCI as a comparable and standardised proxy indicator for comorbidity. With the exception of VRE, CCI was lower in patients than controls, suggesting that the number of previous stays in other health care facilities - based on the assumption that higher morbidity increases the chance of such stays - is not different between controls and colonized patients. VRE on the other hand are usually not as pathogenic as MRSA or MDR-GN, more often causing colonizations rather than infections [30], i.e. instead of killing multimorbid patients, VRE remain colonizing bystanders and are therefore often found in highly morbid patients with increased likelihood of previous hospital stays [31]. These colonizations might thus be a predictor of previous stays in health care facilities.

Only considering cases and controls that never developed an infection can be considered conditioning on the future, as this information is not known at baseline. To circumvent this bias, exposure density sampling is suggested [32]. However, as the infections are very rare within eligible controls, the bias is likely negligible.

Since routine data is used, coding errors may be present. However, according to the department collecting the data, these errors are likely to be random rather than systematic for purposes of our analysis.

Finally, generalizability is another possible limitation, since our data is for one German hospital only. Despite a similar regulatory framework these findings may be different in other German settings and hospitals elsewhere.

While interpreting the results, the definition of the case group as well as the control has to be kept in mind. All patients with an onset infection are excluded. As infections tend to be cost intensive, the selection leads to an observation group which is on average healthier and less expensive. This step is nonetheless necessary in order to isolate the economic burden of MDR colonization, and results may be biased downward, so the conclusion still remains. However, estimates for costs and reimbursements in the literature vary, which is due to heterogeneities in the methodologies and datasets used.

## Conclusions

Taking all strengths and limitations into account, this study demonstrates the importance of accounting for the cost of cases of colonization without infection when analysing the economic burden of antibiotic resistance. The results suggest that MDR bacteria present at hospital admission can add a serious financial burden during a patient’s hospital stay. Since this penalizes the hospitals for events outside their control, a case could be made to classify pre-existing colonization as a type of co-morbidity justifying higher reimbursement.

## Additional file

Additional file 1: Descriptive statistics separated by pathogens. Shows cost, reimbursement, length of stay, age, Charlson comorbidity index, in-hospital mortality and sex for controls as well as for cases separated by pathogen. (DOCX 15 kb)

## Abbreviations

AMR: Antimicrobial resistance
CCI: Charlson comorbidity index
DRG: Diagnosis related groups
GLM: Generalized linear model
HAI: Healthcare-associated infection
ICD-10-GM: International Classification of Diseases 10th revision, German Modification
InEK: Institute for the Payment system in Hospitals
LOS: Length of stay
MDR: Multi-drug resistant
MDR-GN: Multi-resistant gram-negative bacteria
MRSA: Methicillin-resistant *Staphylococcus aureus*
UMCF: University Medical Centre Freiburg
VRE: Vancomycin-resistant *enterococcus*
