# Supplementary material for: In-hospital costs of community-acquired colonization with multidrug-resistant organisms at a German teaching hospital
Source: BMC Health Serv Res. 2018 Sep 26;18:737. doi: 10.1186/s12913-018-3549-0 (PMC6158851; doi:10.1186/s12913-018-3549-0)
Supplement: Supplementary file 1 — Descriptive statistics separated by pathogens. Shows cost, reimbursement, length of stay, age, Charlson comorbidity index, in-hospital mortality and sex for controls as well as for cases separated by pathogen. (DOCX 15 kb) [file 12913_2018_3549_MOESM1_ESM.docx]

Additional file – Descriptive statistics separated by pathogens

|  | 1 | | 2 | | 3 | | 4 | |
| --- | --- | --- | --- | --- | --- | --- | --- | --- |
|  | Actual controls | | MRSA | | VRE | | MDR-GN | |
|  |  |  |  |  |  |  |  |  |
|  | Mean/% | SD | Mean/% | SD | Mean/% | SD | Mean/% | SD |
| Cost, in € | 5504.37 | 8125.41 | 5988.53 | 6061.12 | 7390.47 | 8723.73 | 6176.96 | 8802.77 |
| Reimbursement, in € | 5438.42 | 7023.13 | 6022.39 | 5547.47 | 6579.84 | 7412.31 | 5842.55 | 7538.90 |
| Length of hospital stay, in days | 8.93 | 9.21 | 10.02 | 9.21 | 10.56 | 9.85 | 10.30 | 9.49 |
| Age, in years | 60.76 | 17.38 | 63.13 | 16.98 | 60.95 | 15.86 | 63.59 | 16.84 |
| Charlson comorbidity index | 3.38 | 3.36 | 3.04 | 2.99 | 5.54 | 3.49 | 3.35 | 2.95 |
| In-hospital mortality, % | 2.43 |  | 3.09 |  | 4.68 |  | 2.23 |  |
| Female, % | 44.47 |  | 33.55 |  | 42.53 |  | 50.48 |  |
| Observations | 7917 |  | 453 |  | 1004 |  | 628 |  |

Notes: Column 1 shows all controls chosen for the regression model. Column 2, 3 and 4 show community-onset cases separated for the respective pathogen. There are 90 cases with multiple colonisation.
